# The effect of budesonide combined with bifidobacteria and lactobacilli on lung function and gut microbiota of patients with chronic obstructive pulmonary disease

**DOI:** 10.12669/pjms.40.4.8514

**Published:** 2024

**Authors:** Yan Liu, Feng Ye, Long Ma

**Affiliations:** 1 Yan Liu, Department of Respiratory Medicine, Fengcheng Hospital, Shanghai 201411, P.R. China; 2 Feng Ye, Department of Respiratory Medicine, Fengcheng Hospital, Shanghai 201411, P.R. China; 3 Long Ma, Department of Gastroenterology, Fengcheng Hospital, Shanghai 201411, P.R. China

**Keywords:** Budesonide, Bifidobacteria, Chronic obstructive pulmonary disease, Lactobacilli

## Abstract

**Objective:**

To explore the effects of budesonide combined with Bifidobacteria and Lactobacilli on the lung function and intestinal microbiota of patients with chronic obstructive pulmonary disease (COPD).

**Methods:**

Clinical data of 124 COPD patients admitted to Fengcheng Hospital, Fengxian District, Shanghai from February 2021 to February 2023 were retrospectively analyzed. Patients either received budesonide treatment alone (n=59, control group) or budesonide combined with Bifidobacteria and Lactobacilli (n=65, observation group). Levels of lung function indicators, symptom relief time, gut microbiota levels, and quality of life were compared between the two groups before and after the treatment.

**Results:**

After two weeks of treatment, the improvement of lung function in the observation group was better than that in the control group (*P*<0.05). Compared to budesonide treatment alone, combined budesonide, Bifidobacteria, and Lactobacilli treatment were associated with shorter symptom relief time (*P*<0.05), and with more significant improvement of intestinal microbiota level (*P*<0.05) and the quality of life (*P*<0.05).

**Conclusions:**

Budesonide combined with Bifidobacteria and Lactobacilli can effectively alleviate clinical symptoms, regulate intestinal microbiota, improve lung function and the quality of life of COPD patients.

## INTRODUCTION

Chronic obstructive pulmonary disease (COPD) is a leading cause of world-wide mortality and disability, with a prevalence of 15.70% in men and 9.93% in women.^1^ The main clinical feature of COPD is limited airflow, which is not completely reversible and manifests in varying degrees of difficulty breathing, shortness of breath, expectoration, chronic cough.^1^,^2^ In addition to the pulmonary damage, COPD also affects circulatory system of patients, and in severe cases, may increase morbidity and mortality rates of patients.^2^,^3^ Therefore, early implementation of safe and effective treatment for COPD is of great significance.

Budesonide, a glucocorticoid anti-inflammatory drug, is used currently in clinical practice for the treatment of COPD, and can alleviate clinical symptoms to a certain extent.^4^ However, the overall effect of the drug is not sufficient when used alone.^4^,^5^ Recent studies have found that the onset and progression of COPD has a certain correlation with the intestinal microenvironment.^6^,^7^ Research has shown that microbial colonies are not only distributed in the intestines, but also in the lungs, with a “gut-lung” axis connection.^8^ Disturbance of the gut microbiota not only affects the gastrointestinal immune response but may also have a negative impact on immune states of other organs including lungs.^8^,^9^ Therefore, it is speculated that using probiotics as adjuvant therapy for conventional drugs is beneficial for improving the effectiveness of the treatment.

Bifidobacterium and Lactobacillus are commonly used probiotic preparations in clinical practice, and can effectively regulate the intestinal immune response and correct intestinal microbiota disorders.^10^,^11^ However, there is still limited data on the effectiveness of combining Bifidobacteria and Lactobacilli with budesonide. The aim of this study was to evaluate the value of Bifidobacteria, Lactobacilli and budesonide combination in treating COPD patients.

## METHODS

Clinical data from 124 COPD patients (70 males and 54 females), admitted to Fengcheng Hospital, Fengxian District, Shanghai from February 2021 to February 2023, were retrospectively reviewed. Age of the patients ranged from 51 to 82 years old, with an average of 65.05±8.23 years. The course of the disease was 1-8 years, with an average of 4.09±1.57 years. Of 124 patients, 59 received simple budesonide treatment (Control-group) and 65 patients received budesonide combined with Bifidobacteria and Lactobacilli (Observation-group).

### Ethical Approval

The ethics committee of Fengcheng Hospital approved this study with No. 2021-03 (2021-02-28).

### Inclusion criteria:

- Meets the diagnostic criteria for COPD.^12^
- Age ≥ 18 years old.
- The clinical data is complete.

### Exclusion criteria:

- Individuals with acute or chronic pneumonia, pulmonary tuberculosis, pulmonary edema, and other respiratory diseases.
- Individuals with renal and liver organ dysfunction.
- Individuals with no contraindications to the medications used in this study.
- Individuals with history of chest surgery.
- Individuals with gastrointestinal bleeding, gastric ulcers, and other gastrointestinal diseases.

### Treatment methods:

### Control group

Patients in the control group were treated with budesonide treatment alone. Budesonide suspension (Shanghai Shangyao Xinyi Pharmaceutical Co., Ltd., Specification: 200μg/ puff, National drug approval number: H20010552) - Budesonide 1 mg+physiological saline 2.5 ml, was administered as nebulized inhalation for 15 minutes, twice a day.

### Observation group

Patients in the observation group received budesonide combined with Bifidobacteria and Lactobacilli. The treatment of Budesonide in the observation group was the same as the control group. Bifidobacterium Lactobacillus Triple Viable Tablets (Inner Mongolia Shuangqi Pharmaceutical Co., Ltd., Specification: 0.5 g/tablet, National drug approval number: S19980004) were taken orally 2g/time, three times a day for two weeks.

### Data collection:

Baseline data of patients and the following relevant indicators before and after the treatment were collected:

*Pulmonary function indicators* - forced vital capacity (FVC), forced expiratory volume in one second (FEV1), and peak expiratory flow (PEF) levels were measured using a Master Scope lung function instrument produced by German company Yeger.

*Symptom relief time (day)* - including wheezing relief time, cough relief time, wheezing relief time, and shortness of breath relief time.

### Intestinal microbiota level

One gram of fresh feces from the patient was incubated a 4^0^C for 12 hours and stored in -80^0^C freezer. For the analysis, serial dilutions of the fecal sample were plated on agar plates (Zhengzhou Antu Lvke Biotechnology Co., Ltd, Zhengzhou, China) and cultured at 37^0^C for 36 hours. Bacterial colonies on the plate were counted and bacterial cells were visualized using Gram staining.

### Quality of life

It was assessed using the St. George’s Respiratory Questionnaire (SGRQ).^13^ The SGRQ was calculated using the weighted average method: each question was given different coefficients, called weights, based on previous research, experience, and statistical processing. The greater the impact on quality of life, the higher the weight, and the higher the score. The questionnaire score includes three aspects: respiratory symptom assessment, patient activity, and disease impact. The questionnaire consists of 50 questions, which can be divided into three parts: symptom score (cough, expectoration, wheezing attacks, etc.), activity score (dressing, walking, walking stairs, climbing, etc.), and impact score (sleep, fatigue, worry, fear, etc.). The final quality of life score is the average of the total scores of the above three parts. The score ranges from 0-100, with higher scores indicating a greater impact of the disease on life and a poorer quality of life.

### Statistical Analysis

SPSS25.0 software was used for analysis and processing. The normality of the data was evaluated using the Shapiro-Wilk test. The data of normal distribution were expressed as mean ± standard deviation, and t-test was used for comparison between groups. The data of non-normal distribution were expressed as median and interquartile interval, and analyzed by Mann-Whitney U tests. The counting data were represented as the number of cases, and Chi-square test was used for comparison between groups. When P<0.05, the difference was considered statistically significant.

## RESULTS

A total of 124 patients met the eligibility criteria of this study. The Control-group contained 29 males and 30 females with age range from 51 to 82 years, median age of 65(58, 73) years. The course of the disease in the group was 1-8 years, with median course of 4(2.5) years. The severity of the condition in the group was defined as mild in 22 cases, moderate in 26 cases, and severe in 11 cases. There were 41 males and 24 females in the Observation-group. Age of the group ranged from 51 to 81 years, with median age of 65(58, 71) years. The course of the disease was 1-7 years, with median course of 5(3.5) years. The severity of the condition was defined as mild in 19 cases, moderate in 33 cases, and severe in 13 cases. There was no significant difference in baseline data between the two groups (P>0.05) (Table-I).

**Table-I:** Comparison of baseline data between two groups.

Group	n	Gender (Male/ Female)	Age (years)	Course of the disease (years)	Severity of the condition		

					Mild	Moderate	Severe
Control group	59	29/30	65(58, 70)	4(2, 5)	22	26	11
Observation group	65	41/24	66(56, 73)	5(3, 5)	19	33	13
*χ^2^*/*t*		2.439	-0.388	-1.018	0.929		
*P*		0.118	0.698	0.309	0.629		

There was no significant difference in lung function indicators between the two groups before the treatment (P>0.05). After two weeks of treatment, FVC, FEV1, and PEF in the two groups increased compared to that before the treatment, and was significantly higher in the Observation-group compared to the Control-group (P<0.05) (Table-II).

**Table-II:** Comparison of lung function indicators between two groups.

Time	Group	n	FVC (L)	FEV_1_ (L)	PEF (L/s)
Before treatment	Observation-group	65	1.80±0.27	1.2(1.1, 1.3)	2.32±0.57
	Control-group	59	1.84±0.30	1.1(1.0, 1.3)	2.37±0.60
	t/Z		-0.666	-1.127	-0.500
	P		0.507	0.260	0.618
After treatment	Observation-group	65	2.83±0.41^a^	1.8(1.7, 2.0)^a^	3.32±0.69^a^
	Control-group	59	2.46±0.39^a^	1.6(1.4, 18)^a^	2.82±0.71^a^
	t/Z		5.156	-3.943	3.974
	P		<0.001	<0.001	<0.001

***Note:*** Compared with before treatment in this group,

a P<0.05.

The Observation-group had shorter time for wheezing sound, cough, wheezing, and shortness of breath relief compared to the Control-group (P<0.05) (Table-III).

**Table-III:** Comparison of symptom relief time between two groups (day).

Group	n	Wheezing sound	Cough	Wheezing	Shortness of breath
Observation-group	65	4(3, 5)	5(4, 6)	3(2, 4)	4(3, 5)
Control-group	59	5(4, 5)	7(5, 8)	5(3, 5)	5(5, 6)
*Z*		-3.718	-5.380	-5.653	-5.145
*P*		<0.001	<0.001	<0.001	<0.001

There was no significant difference in the levels of intestinal flora between the two groups before the treatment (P>0.05). After two weeks of treatment, the levels of Enterobacteriaceae and Enterococcus in both groups were lower, and the levels of Lactobacillus and Bifidobacterium were higher than those before the treatment (P<0.05). After the treatment, patients in the Observation-group had significantly lower levels of Enterobacteriaceae and Enterococcus compared to the Control-group, while the levels of Lactobacillus and Bifidobacterium were significantly higher (P<0.05) (Table-IV).

**Table-IV:** Comparison of gut microbiota levels between two groups (LogCFU/g).

Time	Group	n	Enterobacteriaceae	Enterococcus	Lactobacillus	Bifidobacterium
Before treatment	Observation-group	65	9.91±1.84	9.78±1.90	7.14±1.63	7.94±1.74
	Control-group	59	10.31±1.83	9.85±2.10	7.07±1.71	7.78±1.65
	*t*		-1.203	-0.175	0.236	0.521
	*P*		0.231	0.861	0.814	0.603
After treatment	Observation-group	65	6.09±1.63^a^	6.71±1.70^a^	11.82±2.21^a^	11.83±2.19^a^
	Control-group	59	7.32±1.80^a^	7.81±1.80^a^	9.07±1.85^a^	9.49±2.05^a^
	*t*		-4.002	-3.520	7.462	6.128
	*P*		<0.001	0.001	<0.001	<0.001

***Note:*** Compared with before treatment in this group,

a P<0.05.

There was no significant difference in SGRQ scores between the two groups before the treatment (P>0.05). After two weeks of treatment, the disease impact, symptoms, and activity scores of the two groups decreased compared to those before the treatment (P<0.05). Patients in the Observation-group had significantly lower post-treatment scores compared to the Control-group (P<0.05) (Table-V).

**Table-V:** Comparison of SGRQ scores for quality of life between two groups (score).

Time	Group	n	Disease impact	symptom	Activity
Before treatment	Observation-group	65	12(11,14)	23(20,25)	18(15, 20)
	Control-group	59	12(9, 14)	21(18, 25)	19(15, 20)
	Z		-0.320	-1.524	-0.900
	P		0.749	0.128	0.368
After treatment	Observation-group	65	9(8, 11)^a^	15(12, 17)^a^	14(12, 15)^a^
	Control-group	59	10(9, 12)^a^	17(14, 20)^a^	17(13, 18)^a^
	Z		-2.200	-4.660	-4.272
	P		0.028	<0.001	<0.001

***Note:*** Compared with before treatment in this group,

a P<0.05.

## DISCUSSION

The results of this study indicated that budesonide combined with Bifidobacteria and Lactobacilli has a high application value in the treatment of COPD. Compared to budesonide alone, the combined treatment was more effective in alleviating clinical symptoms, reducing the impact of the disease and markedly improving the quality of life of COPD patients.

Pei C et al^14^ confirmed that comprehensive intervention with probiotics based on conventional drug therapy for COPD can further alleviate patients’ clinical symptoms and improve the overall therapeutic effect. Karim A et al^15^ also pointed out that adding probiotics to the routine treatment regimen of COPD patients not only improves their walking speed and grip strength, but also has a positive effect on downregulating the expression of C-reactive proteins and reducing the degree of inflammation in the body. Similarly, Hua JL et al^16^ also reported that oral probiotics can effectively reduce the frequency of acute exacerbation of COPD and correlate with better disease outcomes. Moreover, Carvalho JL et al^17^ confirmed that oral administration of probiotic Lactobacillus rhamnosus can alleviate clinical symptoms of COPD induced by cigarette smoke in C57Bl/6 mice. Based on these results, the authors speculated that probiotics play an important role in maintaining and regulating the balance between anti-inflammatory and pro-inflammatory cytokines in bronchial epithelial cells after cigarette smoke exposure, and can become an important tool for alleviating pulmonary inflammatory response in COPD patients.^16^,^17^ The results of this study are consistent with the above observations.

The pathogenic factors of COPD are complex and are mainly related to the inhalation of harmful gases leading to inflammatory responses or an increase in inflammatory cells in the lungs, which in turn leads to the inflammatory reactions in the airways, promotes the formation of fibrous tissue, and subsequently causes structural abnormalities in the lungs.^3^ Gut microbiota plays an important role in immune system regulation, nutrient absorption, and intestinal mucosal protection.^8^,^9^ Bowerman KL et al^18^ identified a number of risk factors in the pathogenesis and progression of COPD that may lead to intestinal microbiota imbalance and intestinal microenvironment disturbance. Harmful bacterial invasion may result in intestinal bacteria breaking through the intestinal mucosa and reaching the mesenteric lymph nodes or distant organs, which can lead to a massive release of inflammatory mediators, damaging the intestinal mucosa, and further exacerbating the inflammatory response and disease.^9^,^18^

Li N et al^19^ have shown that the incidence of intestinal microbiota disorder in COPD patients may be as high as 40%. Imbalance of intestinal microbiota may accelerate the onset and progression of COPD by reducing the body’s immune system, promoting the translocation of intestinal pathogenic bacteria, and generating endotoxins, and these factors are causal and mutually influencing.^8^,^9^,^19^ The results of our study also confirmed that combining budesonide with Bifidobacteria and Lactobacilli in the treatment of COPD can help regulate the intestinal microbiota status and improve the effectiveness of the treatment. Bifidobacterium and Lactobacillus are probiotics composed of fecal Enterococcus, Bifidobacterium, Lactobacillus acidophilus and other bacteria.^10^ Oral administration of them can promote the colonization of probiotics in the intestine, increase the number of normal flora, restore the affected balance of gastrointestinal flora, strengthen intestinal defense, and maintain the overall integrity of the gastrointestinal tract.^10^,^11^ Furthermore, Ananya FN et al^20^ found that exogenous probiotic preparations, taken as supplements, can stimulate the proliferation of intestinal epithelial cells, lower intestinal mucosal permeability, inhibit intestinal endotoxin secretion, promote the uptake and absorption of nutrients, and protect the intestinal mucosa. Saint-Criq V et al^21^ also showed that probiotic preparations can promote the proliferation of probiotic microorganisms in the intestine, reduce excessive reproduction of pathogenic bacteria, and maintain the balance of intestinal microbiota, which plays an important role in improving treatment efficiency.^20^,^21^

In addition, Gao et al. have found budesonide combined with noninvasive ventilation could improve the quality of life in patients with COPD.^22^ However, few studies have investigated the effect of Bifidobacteria and Lactobacilli on the quality of life in patients with COPD. This study confirmed that the quality of life of the patients improved after the combined therapy, ecause the alleviation of clinical symptoms and the improvement of lung function and intestinal microbiota would consequently improve the quality of life.

### Limitations

This is a single center retrospective analysis, with a small sample size and no follow-up observation of the patients. It cannot be ruled out that the patients have used other medications during the treatment process. Further robust randomized, double-blind, and prospective clinical trials are needed to validate the results of this study.

## CONCLUSION

The treatment of COPD with budesonide combined with Bifidobacteria and Lactobacilli can effectively alleviate clinical symptoms, regulate intestinal microbiota, improve lung function, and help improve patients’ quality of life.

### Authors’ contributions:

**YL:** Conceived and designed the study.

**FY** and **LM:** Collected the data and performed the analysis.

**YL:** Was involved in the writing of the manuscript and is responsible for the integrity of the study.

All authors have read and approved the final manuscript.
